# Atlanta Medical College

**Published:** 1861-10

**Authors:** 


### Atlanta Medical College.

To the numerous inquiries in regard to the probability of having a Course of Lectures in 1862, we answer that the Faculty have determined to open the Session as usual, the first Monday in May.

This has been made more necessary, during these times of Governmental troubles, by the suspension of nearly all the winter Schools of Medicine the present season. Our Course for 1861 was cut short several weeks by the enlistment, in the Army, of both Faculty and Students to the extent of making it necessary to do so; and though half the Faculty have been engaged in military service ever since, and will, most of them, so continne through the winter; all will make such arrangements as are necessary to insure their filling the several chairs promptly and perfectly. While the Faculty have thus determined, they know that the public mind is completely absorbed with our National troubles, and that the energies of all should be directed to the army. We feel that the promotion of that science which is brought into requisition so constantly—so far as it can be done consistently with the service—is not a departure from the principle just announced. We say let every private interest give way to the great cause of our National independence; and also of every public enterprise, though it may be an auxiliary to the country's cause remotely, if any considerable direct injury is sustained thereby. Then with the knowledge of all these facts, and a feeling sense of our duty to the country, we have determined to resume the exercises of the College at the usual period. Those who feel it to be their interest, and not inconsistent with their duty, to attend, we will be pleased to see; and will afford them *all* the facilities for learning heretofore found in the Institution. VOLUNTEER.

